# Supplementary material for: A machine learning approach to support triaging of primary versus secondary headache patients using complete blood count
Source: PLoS One. 2023 Mar 6;18(3):e0282237. doi: 10.1371/journal.pone.0282237 (PMC9987784; doi:10.1371/journal.pone.0282237)
Supplement: S1 Table — (DOCX) [file pone.0282237.s001.docx]

## **S1 Table.**

| **Read Code** | **Description** |
| --- | --- |
| **Migraine** | |
| Fyu5300 | Other migraine |
| F26z.00 | Migraine NOS |
| F26yz00 | Other forms of migraine NOS |
| F26y300 | Complicated migraine |
| F26y111 | Moebius' ophthalmoplegic migraine |
| F26y100 | Ophthalmoplegic migraine |
| F26y000 | Hemiplegic migraine |
| F26y.00 | Other forms of migraine |
| F262z00 | Migraine variant NOS |
| F262800 | Migraine induced by estrogen contraceptive |
| F262400 | Ophthalmic migraine |
| F262300 | Basilar migraine |
| F262200 | Abdominal migraine |
| F262.00 | Migraine variants |
| F261z00 | Common migraine NOS |
| F261000 | Atypical migraine |
| F261.11 | Migraine without aura |
| F261.00 | Common migraine |
| F260.11 | Migraine with aura |
| F260.00 | Classical migraine |
| F26..00 | Migraine |
| **Tension** | |
| E278100 | Tension headache |
| F262600 | Tension type headache |
| F262B00 | Chronic tension-type headache |
| F262A00 | Frequent episodic tension-type headache |
| F262900 | Infrequent episodic tension-type headache |
| **Cluster headache** | |
| F262000 | Cluster headache |
