# Supplementary material for: A machine learning approach to support triaging of primary versus secondary headache patients using complete blood count
Source: PLoS One. 2023 Mar 6;18(3):e0282237. doi: 10.1371/journal.pone.0282237 (PMC9987784; doi:10.1371/journal.pone.0282237)
Supplement: S2 Table — (DOCX) [file pone.0282237.s002.docx]

**S2 Table.**

| **Medical Code** | **Read Code** | **Description** |
| --- | --- | --- |
| **Migraine** | | |
| 63055014 | F26..00 | Migraine |
| 7596016 | F260.00 | Classical migraine |
| 7595017 | F260.11 | Migraine with aura |
| 93295014 | F261.00 | Common migraine |
| 93294013 | F261.11 | Migraine without aura |
| 93296010 | F261000 | Atypical migraine |
| 297336013 | F261z00 | Common migraine NOS |
| 297337016 | F262.00 | Migraine variants |
| 126030010 | F262200 | Abdominal migraine |
| 138248010 | F262300 | Basilar migraine |
| 158460019 | F262400 | Ophthalmic migraine |
| 297351016 | F262z00 | Migraine variant NOS |
| 1746951000000114 | F262800 | Migraine induced by estrogen contraceptive |
| 297352011 | F26y.00 | Other forms of migraine |
| 98501012 | F26y000 | Hemiplegic migraine |
| 82623012 | F26y100 | Ophthalmoplegic migraine |
| 700211000006115 | F26y111 | Moebius' ophthalmoplegic migraine |
| 297354012 | F26y300 | Complicated migraine |
| 297355013 | F26yz00 | Other forms of migraine NOS |
| 297356014 | F26z.00 | Migraine NOS |
| 299338017 | Fyu5300 | Other migraine |
| **Tension** | | |
| 1786078018 | E278100 | Tension headache |
| 359201000006116 | F262600 | Tension type headache |
| 1754211000000113 | F262900 | Infrequent episodic tension-type headache |
| 1756651000000117 | F262A00 | Frequent episodic tension-type headache |
| 345361012 | F262B00 | Chronic tension-type headache |
| **Cluster headache** | | |
| 297338014 | F262000 | Cluster headache |
| 2838283014 | F262D00 | Paroxysmal hemicrania |
| 1757331000000118 | F262E00 | Trigeminal autonomic cephalalgia |
