# Supplementary material for: A machine learning approach to support triaging of primary versus secondary headache patients using complete blood count
Source: PLoS One. 2023 Mar 6;18(3):e0282237. doi: 10.1371/journal.pone.0282237 (PMC9987784; doi:10.1371/journal.pone.0282237)
Supplement: S3 Table — (DOCX) [file pone.0282237.s003.docx]

**S3 Table.**

| **Read Code** | **Description** |
| --- | --- |
| **Ischemic stroke** | |
| G66..12 | Stroke unspecified |
| G64..13 | Stroke due to cerebral arterial occlusion |
| G66..00 | Stroke and cerebrovascular accident unspecified |
| **Cerebral venous thrombosis** | |
| L417.00 | Obstetric cerebral venous thrombosis |
| L417000 | Cerebral venous thrombosis in pregnancy |
| L417100 | Cerebral venous thrombosis in the puerperium |
| G676000 | Cerebral infarct due cerebral venous thrombosis, nonpyogenic |
| G67B.00 | Reversible cerebral vasoconstriction syndrome |
| **Hemorrhage** | |
| G60..00 | Subarachnoid hemorrhage |
| G601.00 | Subarachnoid hemorrhage from carotid siphon and bifurcation |
| G602.00 | Subarachnoid hemorrhage from middle cerebral artery |
| G603.00 | Subarachnoid hemorrhage from anterior communicating artery |
| G604.00 | Subarachnoid hemorrhage from posterior communicating artery |
| G605.00 | Subarachnoid hemorrhage from basilar artery |
| G606.00 | Subarachnoid hemorrhage from vertebral artery |
| G60z.00 | Subarachnoid hemorrhage NOS |
| G61..00 | Intracerebral hemorrhage |
| G61..11 | Cerebrovascular accident due to intracerebral hemorrhage |
| G617.00 | Intracerebral hemorrhage, intraventricular |
| G618.00 | Intracerebral hemorrhage, multiple localized |
| G61z.00 | Intracerebral hemorrhage NOS |
| G62..00 | Other and unspecified intracranial hemorrhage |
| G621.00 | Subdural hemorrhage - nontraumatic |
| G623.00 | Subdural hemorrhage NOS |
| G62z.00 | Intracranial hemorrhage NOS |
| G680.00 | Sequelae of subarachnoid hemorrhage |
| G682.00 | Sequelae of other nontraumatic intracranial hemorrhage |
| Gyu6000 | Subarachnoid hemorrhage from other intracranial arteries |
| Gyu6100 | Other subarachnoid hemorrhage |
| Q200012 | Intracranial hemorrhage in fetus or newborn |
| Q200100 | Subdural hemorrhage unspecified, due to birth trauma |
| Q412.00 | Perinatal subarachnoid hemorrhage |
| Q412000 | Subarachnoid hemorrhage due to birth injury |
| Q417.00 | Intracranial nontraumatic hemorrhage of fetus and newborn |
| S62..13 | Subdural hemorrhage following injury |
| S622.00 | Closed traumatic subdural hemorrhage |
| S623.00 | Open traumatic subdural hemorrhage |
| S627.00 | Traumatic subarachnoid hemorrhage |
| S628.00 | Traumatic subdural hemorrhage |
| **Arteritis** | |
| G755z00 | Giant cell arteritis NOS |
| Nyu4100 | Other giant cell arteritis |
| N200.00 | Giant cell arteritis with polymyalgia rheumatica |
| G755.00 | Giant cell arteritis |
| **Angiitis** | |
| G731z00 | Thromboangiitis obliterans NOS |
| G75A.00 | Microscopic polyangiitis |
| G752.00 | Hypersensitivity angiitis |
| G752z00 | Hypersensitivity angiitis NOS |
| G754.11 | Granulomatosis with polyangiitis |
| G750.11 | Necrotizing angiitis |
| G731.00 | Thromboangiitis obliterans |
