# Supplementary material for: A machine learning approach to support triaging of primary versus secondary headache patients using complete blood count
Source: PLoS One. 2023 Mar 6;18(3):e0282237. doi: 10.1371/journal.pone.0282237 (PMC9987784; doi:10.1371/journal.pone.0282237)
Supplement: S4 Table — CVA, cerebrovascular accident; NOS, not otherwise specified. (DOCX) [file pone.0282237.s004.docx]

**S4 Table.**

| **Medical Code** | **Read Code** | **Description** |
| --- | --- | --- |
| **Ischemic stroke** | | |
| 411416011 | L440.12 | Stroke in the puerperium |
| 426321000006116 | Gyu6C00 | Sequelae of stroke, not specified as hemorrhage or infarction |
| 149571000006118 | G68X.00 | Sequelae of stroke, not specified as hemorrhage or infarction |
| 300366019 | G664.00 | Cerebellar stroke syndrome |
| 524511000006116 | G663.00 | Brain stem stroke syndrome |
| 884531000006117 | G66..99 | Stroke |
| 884521000006115 | G66..98 | Stroke/CVA - undefined |
| 122401000006115 | G66..12 | Stroke unspecified |
| 405339016 | G66..00 | Stroke and CVA unspecified |
| 122361000006113 | G64..13 | Stroke due to cerebral arterial occlusion |
| 122371000006118 | G61..12 | Stroke due to intracerebral hemorrhage |
| **Cerebral venous thrombosis** | | |
| 1729331000006116 |  | Cerebral venous thrombosis |
| 1859271000006112 |  | Reversible cerebral vasoconstriction syndrome |
| 542251000006112 | G676000 | Cerebral infarct due cerebral venous thrombosis, nonpyogenic |
| 2262511000000116 | G67B.00 | Reversible cerebral vasoconstriction syndrome |
| 307780013 | L417.00 | Obstetric cerebral venous thrombosis |
| 307781012 | L417000 | Cerebral venous thrombosis in pregnancy |
| 307782017 | L417100 | Cerebral venous thrombosis in the puerperium |
| **Hemorrhage** | | |
| 481028017 | G60..00 | Subarachnoid hemorrhage |
| 300244012 | G601.00 | Subarachnoid hemorrhage from carotid siphon and bifurcation |
| 123511000006114 | G602.00 | Subarachnoid hemorrhage from middle cerebral artery |
| 123481000006118 | G603.00 | Subarachnoid hemorrhage from anterior communicating artery |
| 123521000006118 | G604.00 | Subarachnoid hemorrhage from posterior communicating artery |
| 123491000006115 | G605.00 | Subarachnoid hemorrhage from basilar artery |
| 300253017 | G606.00 | Subarachnoid hemorrhage from vertebral artery |
| 300257016 | G60z.00 | Subarachnoid hemorrhage NOS |
| 744901000006114 | G61..00 | Intracerebral hemorrhage |
| 605471000006112 | G61..11 | CVA - cerebrovascular accident due to intracerebral hemorrhage |
| 300277011 | G617.00 | Intracerebral hemorrhage, intraventricular |
| 746571000006116 | G618.00 | Intracerebral hemorrhage, multiple localized |
| 300287010 | G61z.00 | Intracerebral hemorrhage NOS |
| 300290016 | G62..00 | Other and unspecified intracranial hemorrhage |
| 300294013 | G621.00 | Subdural hemorrhage - nontraumatic |
| 2534198011 | G623.00 | Subdural hemorrhage NOS |
| 300298011 | G62z.00 | Intracranial hemorrhage NOS |
| 300406018 | G680.00 | Sequelae of subarachnoid hemorrhage |
| 149551000006111 | G682.00 | Sequelae of other nontraumatic intracranial hemorrhage |
| 300935019 | Gyu6000 | Subarachnoid hemorrhage from other intracranial arteries |
| 300936018 | Gyu6100 | Other subarachnoid hemorrhage |
| 769281000006119 | Q200012 | Intracranial hemorrhage in fetus or newborn |
| 316140013 | Q200100 | Subdural hemorrhage unspecified, due to birth trauma |
| 480952017 | Q412.00 | Perinatal subarachnoid hemorrhage |
| 316519019 | Q412000 | Subarachnoid hemorrhage due to birth injury |
| 316553015 | Q417.00 | Intracranial nontraumatic hemorrhage of fetus and newborn |
| 320836010 | S62..13 | Subdural hemorrhage following injury |
| 320752018 | S622.00 | Closed traumatic subdural hemorrhage |
| 320771016 | S623.00 | Open traumatic subdural hemorrhage |
| 391043019 | S627.00 | Traumatic subarachnoid hemorrhage |
| 320835014 | S628.00 | Traumatic subdural hemorrhage |
| **Arteritis** | | |
| 2534171012 | G755.00 | Giant cell arteritis |
| 300584016 | G755z00 | Giant cell arteritis NOS |
| 359513016 | N200.00 | Giant cell arteritis with polymyalgia rheumatica |
| 312576015 | Nyu4100 | Other giant cell arteritis |
| 1779388019 | G755100 | Temporal arteritis |
| 5141661000006114 |  | Giant cell arteritis without polymyalgia rheumatica |
| **Angiitis** | | |
| 87208017 | G731.00 | Thromboangiitis obliterans |
| 300503012 | G731z00 | Thromboangiitis obliterans NOS |
| 1783725016 | G750.11 | Necrotizing angiitis |
| 100606014 | G752.00 | Hypersensitivity angiitis |
| 300577012 | G752z00 | Hypersensitivity angiitis NOS |
| 2955624019 | G754.11 | Granulomatosis with polyangiitis |
| 2157271000000115 | G75A.00 | Microscopic polyangiitis |
