# Supplementary material for: A machine learning approach to support triaging of primary versus secondary headache patients using complete blood count
Source: PLoS One. 2023 Mar 6;18(3):e0282237. doi: 10.1371/journal.pone.0282237 (PMC9987784; doi:10.1371/journal.pone.0282237)
Supplement: S5 Table — (DOCX) [file pone.0282237.s005.docx]

**S5 Table.**

| **Read Code** | **Description** |
| --- | --- |
| 1B1G.00 | Headache |
| 1B1G.11 | C/O - a headache |
| 1B1G000 | Sinus headache |
| 1B1G100 | Viral headache |
| 1BA..00 | Headache site |
| 1BA2.00 | Generalized headache |
| 1BA3.00 | Unilateral headache |
| 1BA4.00 | Bilateral headache |
| 1BA5.00 | Frontal headache |
| 1BA6.00 | Occipital headache |
| 1BA7.00 | Parietal headache |
| 1BA8.00 | Temporal headache |
| 1BA9.00 | Sinus headache |
| 1BAZ.00 | Headache site NOS |
| 1BB..00 | Headache character |
| 1BB1.00 | Aching headache |
| 1BB2.00 | Throbbing headache |
| 1BB3.00 | Shooting headache |
| 1BB4.00 | Morning headache |
| 1BBZ.00 | Headache character NOS |
| 1B1G.12 | Cephalgia |
