# Supplementary material for: A machine learning approach to support triaging of primary versus secondary headache patients using complete blood count
Source: PLoS One. 2023 Mar 6;18(3):e0282237. doi: 10.1371/journal.pone.0282237 (PMC9987784; doi:10.1371/journal.pone.0282237)
Supplement: S6 Table — (DOCX) [file pone.0282237.s006.docx]

**S6 Table.**

| **Medical Code** | **Read Code** | **Description** |
| --- | --- | --- |
| 142901000006114 | 1B1G000 | Sinus headache |
| 1634018 | 1BA6.00 | Occipital headache |
| 166489012 | 1BA3.00 | Unilateral headache |
| 252948015 | 1B1G100 | Viral headache |
| 253103010 | 1BA..00 | Headache site |
| 253105015 | 1BA2.00 | Generalized headache |
| 253109014 | 1BA4.00 | Bilateral headache |
| 253115014 | 1BA7.00 | Parietal headache |
| 253116010 | 1BAZ.00 | Headache site NOS |
| 253117018 | 1BB..00 | Headache character |
| 253118011 | 1BB1.00 | Aching headache |
| 253119015 | 1BB2.00 | Throbbing headache |
| 253120014 | 1BB3.00 | Shooting headache |
| 253121013 | 1BB4.00 | Morning headache |
| 253123011 | 1BBZ.00 | Headache character NOS |
| 397993014 | 1BA5.00 | Frontal headache |
| 407069017 | 1B1G.11 | C/O - a headache |
| 41990019 | 1B1G.00 | Headache |
| 41994011 | 1B1G.12 | Cephalgia |
| 69073019 | 1BA8.00 | Temporal headache |
| 9300010 | 1BA9.00 | Sinus headache |
