# Supplementary material for: A machine learning approach to support triaging of primary versus secondary headache patients using complete blood count
Source: PLoS One. 2023 Mar 6;18(3):e0282237. doi: 10.1371/journal.pone.0282237 (PMC9987784; doi:10.1371/journal.pone.0282237)
Supplement: S7 Table — (DOCX) [file pone.0282237.s007.docx]

**S7 Table.**

| **Medical Code** | **Read Code** | **Ent type Code** | **Description** |
| --- | --- | --- | --- |
| 17 | 426..00 | 194 | Red blood cell count |
| 7 | 42P..00 | 189 | Platelets |
| 13817, 26947, 15 | 42H..11  42H7.00 | 207 | White blood cell count |
| 18 | 42J..00 | 184 | Neutrophil count |
| 19 | 42M..00 | 208 | Lymphocyte count |
| 21 | 42N..00 | 183 | Monocyte count |
| 22 | 42K..00 | 168 | Eosinophil count |
| 25 | 42L..00 | 313 | Basophil count |
| 10 | 42A..00 | 182 | Mean corpuscular volume |
| 4 | 423..00 | 173 | Hemoglobin |
