# Supplementary material for: A machine learning approach to support triaging of primary versus secondary headache patients using complete blood count
Source: PLoS One. 2023 Mar 6;18(3):e0282237. doi: 10.1371/journal.pone.0282237 (PMC9987784; doi:10.1371/journal.pone.0282237)
Supplement: S8 Table — (DOCX) [file pone.0282237.s008.docx]

**S8 Table.**

| **Medical Code** | **Read code** | **Description** |
| --- | --- | --- |
| 183211000006119 | 426..00 | Red blood cell count |
| 102928018 | 42P..00 | Platelets |
| 98461000006116 | 42H..00 | White blood cell count |
| 51268016 | 42J..00 | Neutrophil count |
| 124165010 | 42M..00 | Lymphocyte count |
| 112567012 | 42N..00 | Monocyte count |
| 119575019 | 42K..00 | Eosinophil count |
| 70669012 | 42L..00 | Basophil count |
| 714931000006112 | 42A..00 | Mean corpuscular volume |
| 813551000006113 | 423..00 | Hemoglobin |
