# Supplementary material for: A machine learning approach to support triaging of primary versus secondary headache patients using complete blood count
Source: PLoS One. 2023 Mar 6;18(3):e0282237. doi: 10.1371/journal.pone.0282237 (PMC9987784; doi:10.1371/journal.pone.0282237)
Supplement: S9 Table — (DOCX) [file pone.0282237.s009.docx]

**S9 Table.**

| **Medical Code** | **Read Code** | **Description** |
| --- | --- | --- |
| 94046 | 9b0E.00 | Follow-up/routine visit note |
| 28647 | 9b0R.00 | Mail from patient |
