# Supplementary figures and images for: A machine learning approach to support triaging of primary versus secondary headache patients using complete blood count
Source: PLoS One. 2023 Mar 6;18(3):e0282237. doi: 10.1371/journal.pone.0282237 (PMC9987784; doi:10.1371/journal.pone.0282237)

## **S1 Fig.**

**A**


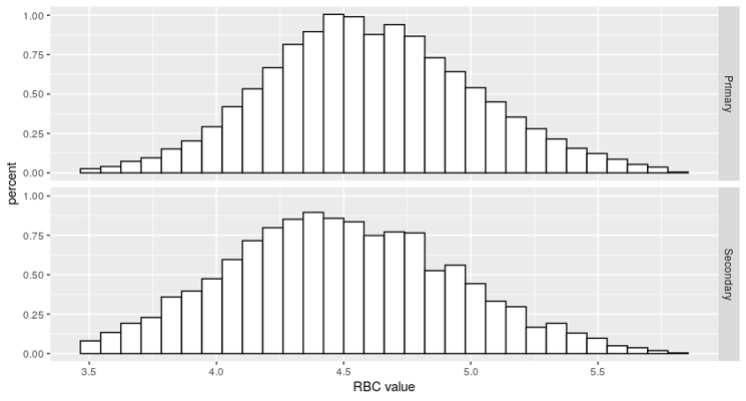


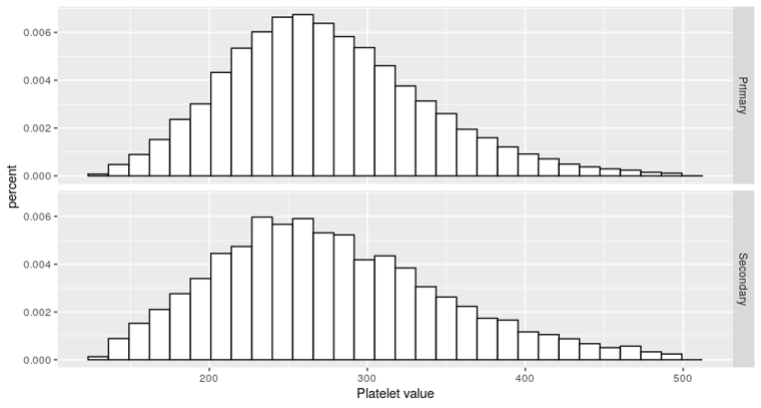


**B**


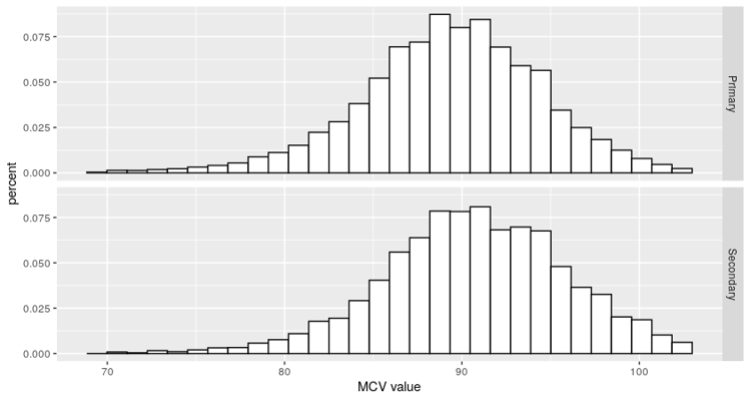


**C**


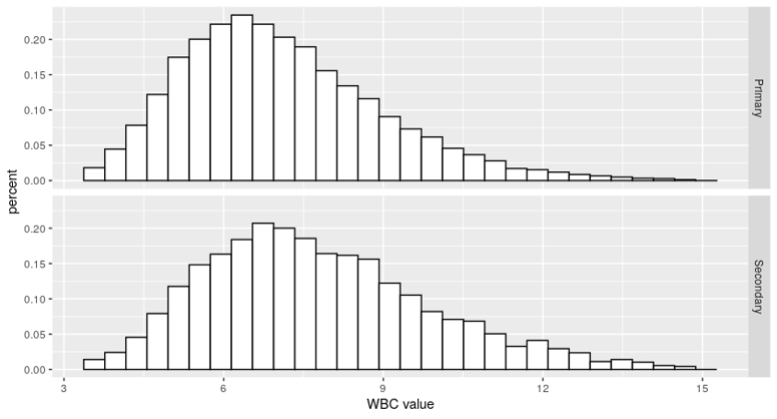


**D**


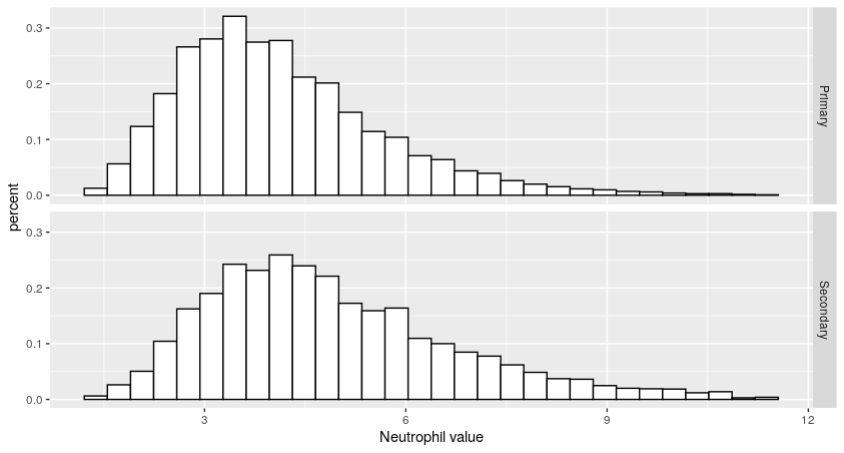


**F**

**E**


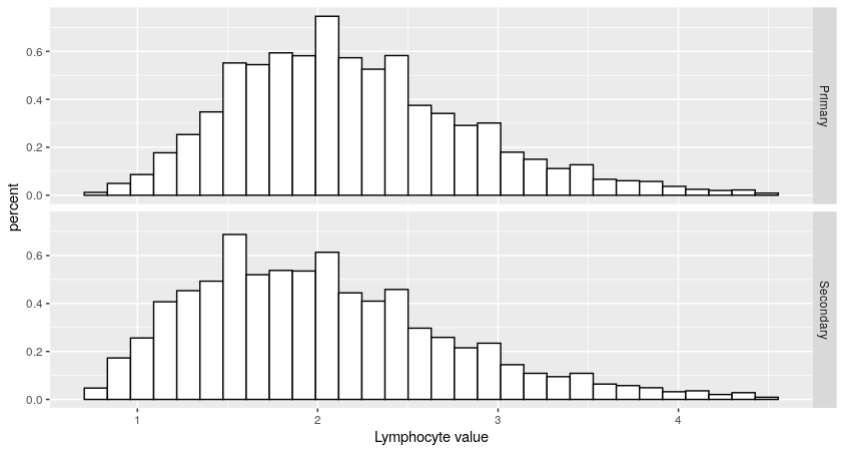


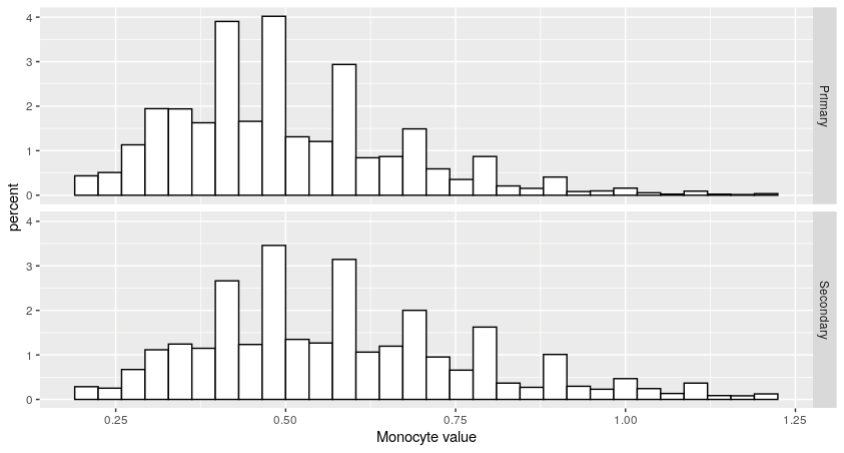


**G**


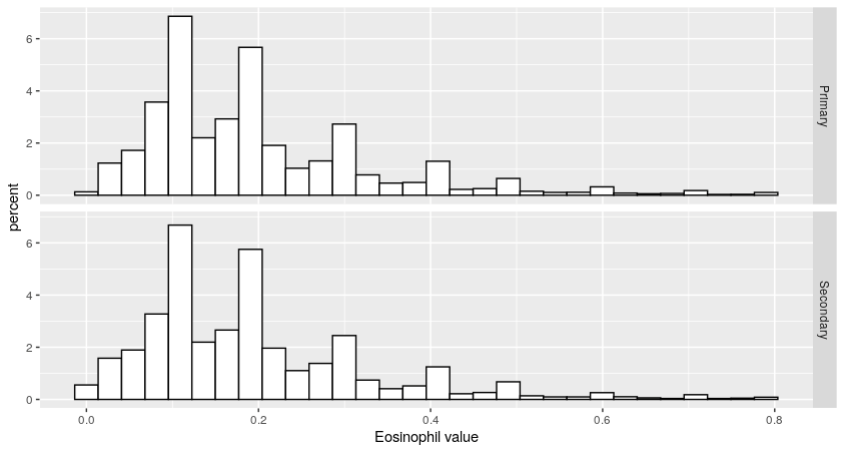


**H**


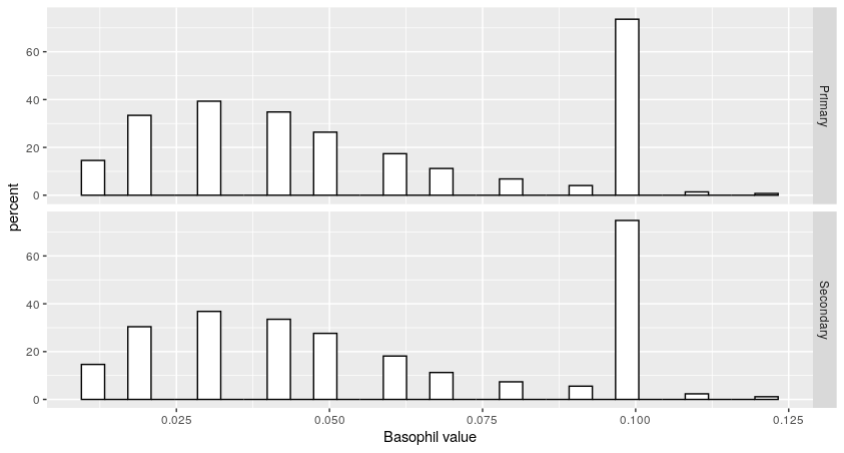


**I**


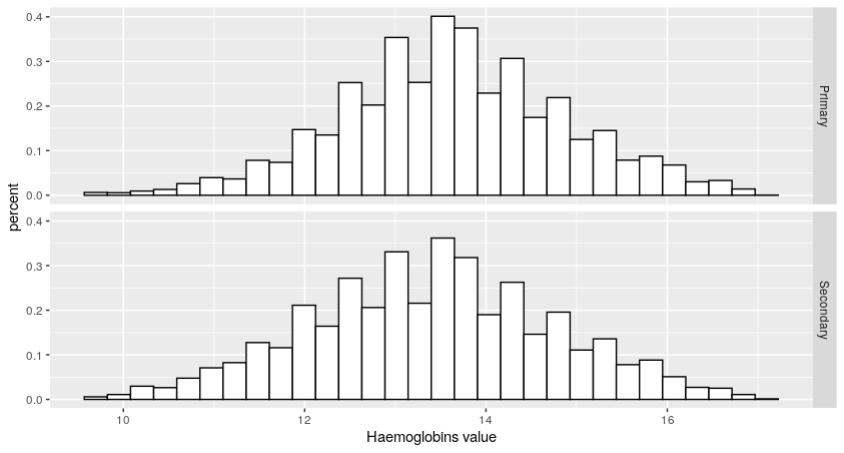


**J**

Supplement: S1 Fig — Distribution of the following 10 parameters from CBC test results by headache group (data cleaned by removing the extreme values of blood test results): A. RBC count (1012/L), B. platelet count (109/L), C. MCV (fL), D. WBC count (109/L), E. neutrophil count (109/L), F. lymphocyte count (109/L), G. monocyte count (109/L), H. eosinophil count (109/L), I. basophil count (109/L), J. hemoglobin (g/dL). CBC, complete blood count; MCV, mean corpuscular volume; RBC, red blood cell; WBC, white blood cell. (DOCX) [file pone.0282237.s011.docx]

## **S2 Fig.**

**A**


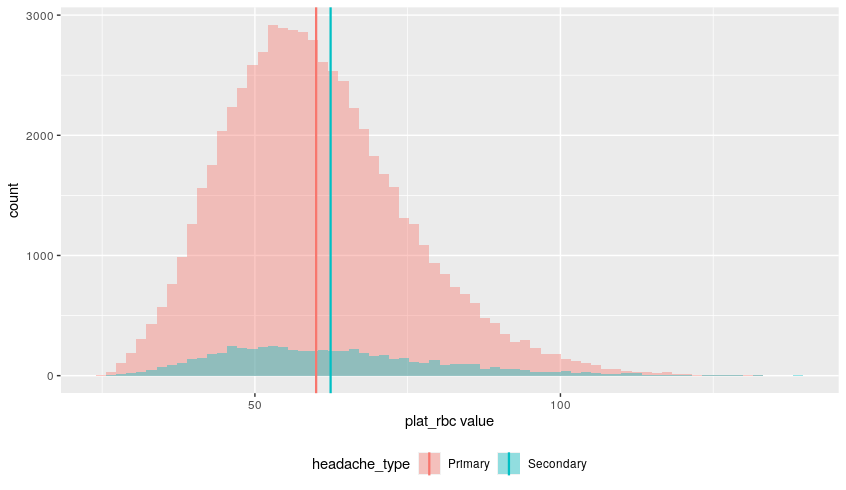


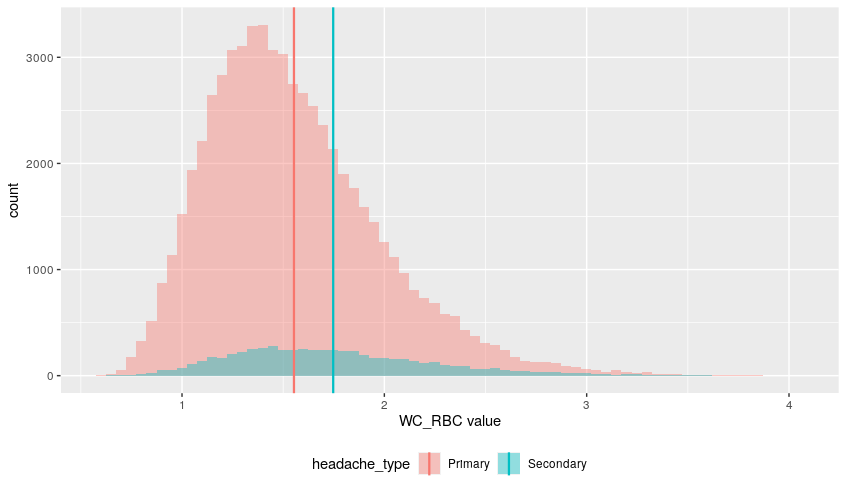


**B**

**C**


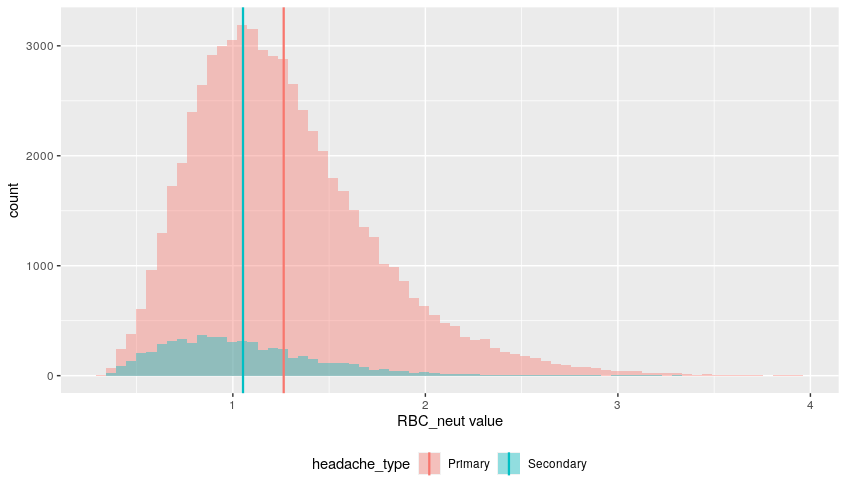


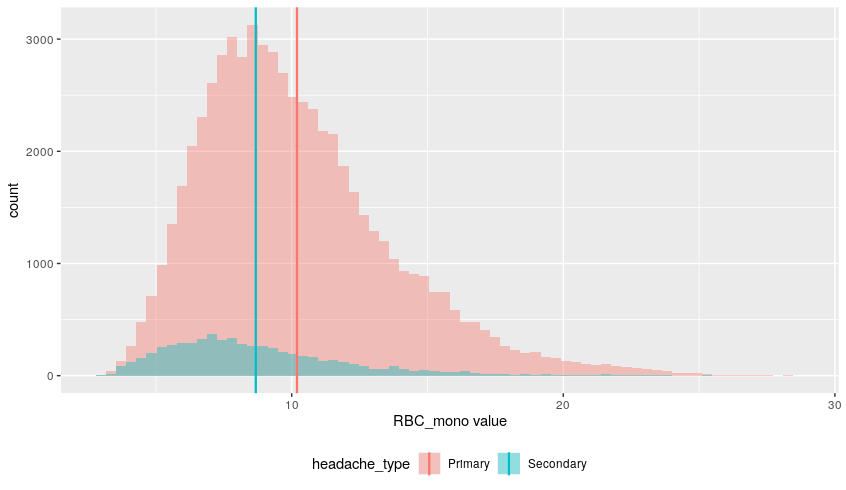


**D**

**E**


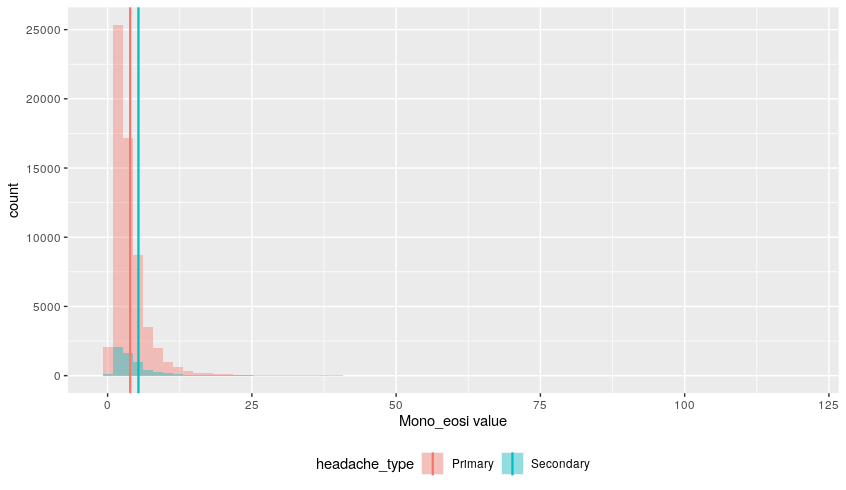


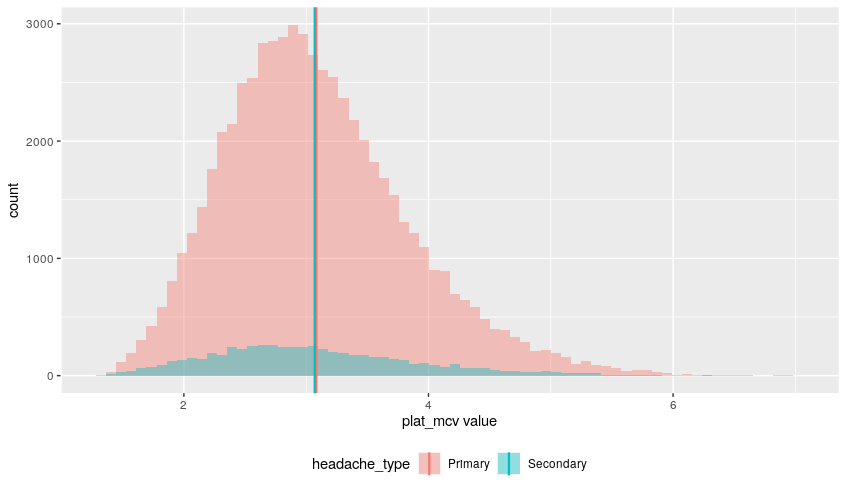


**F**

**G**


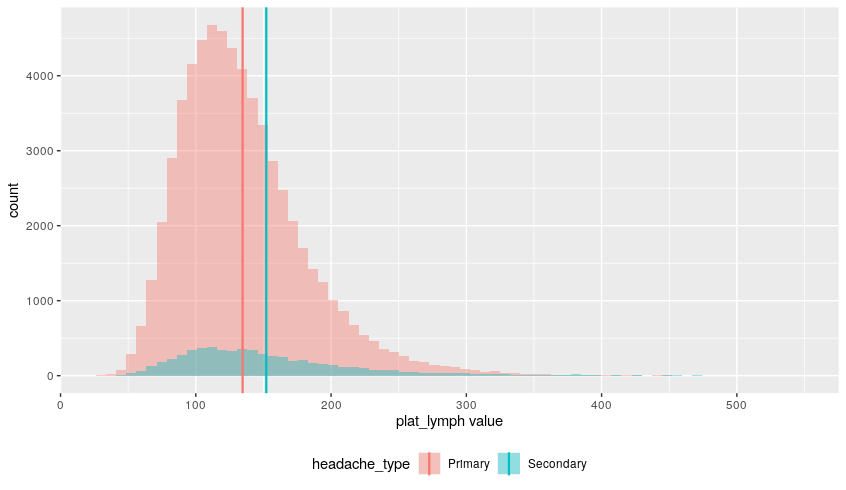


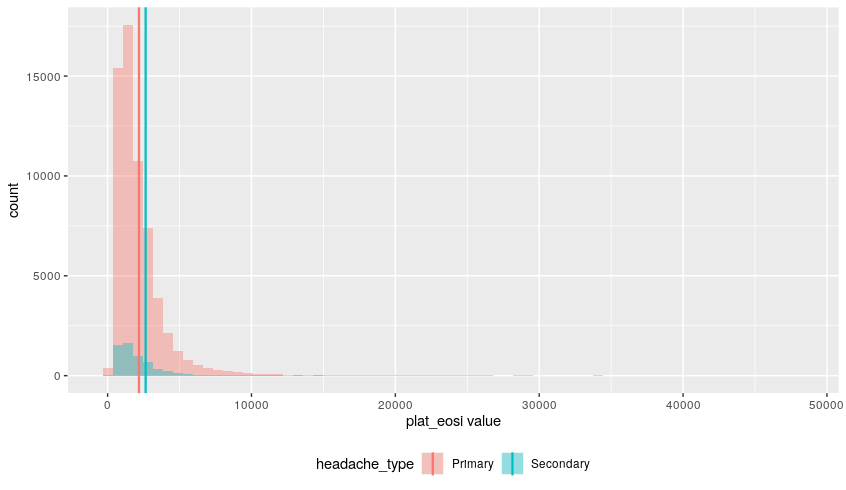


**H**

**I**


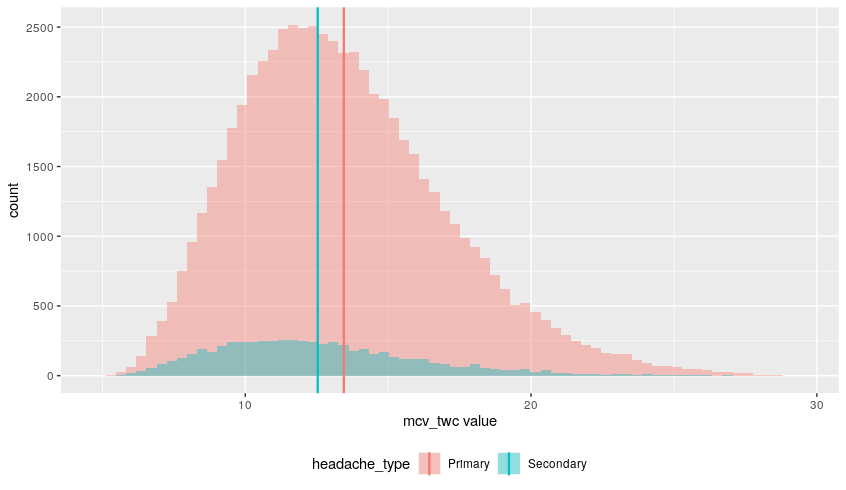


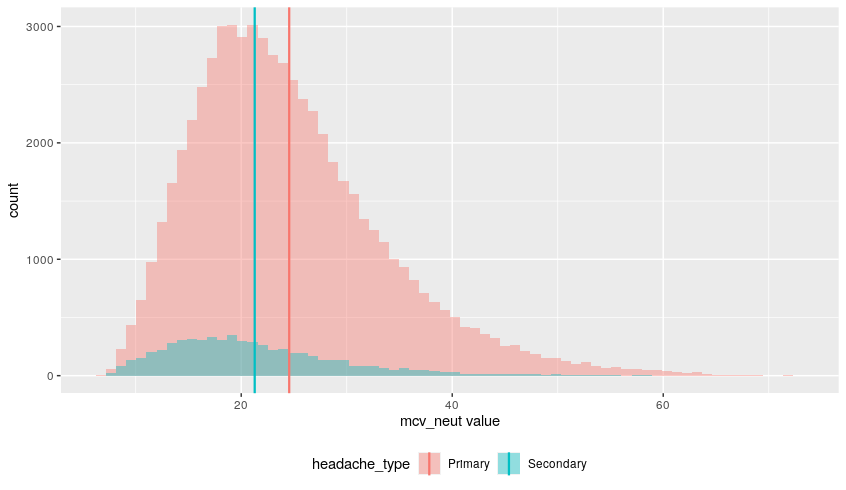


**J**

**K**


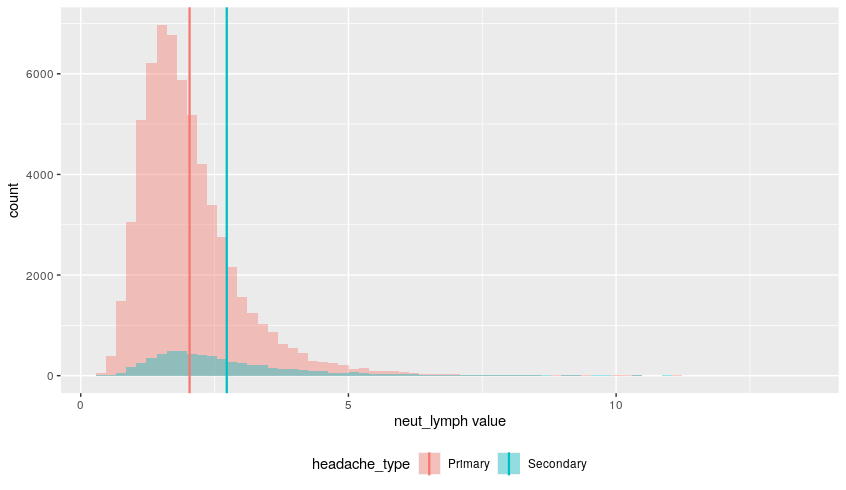


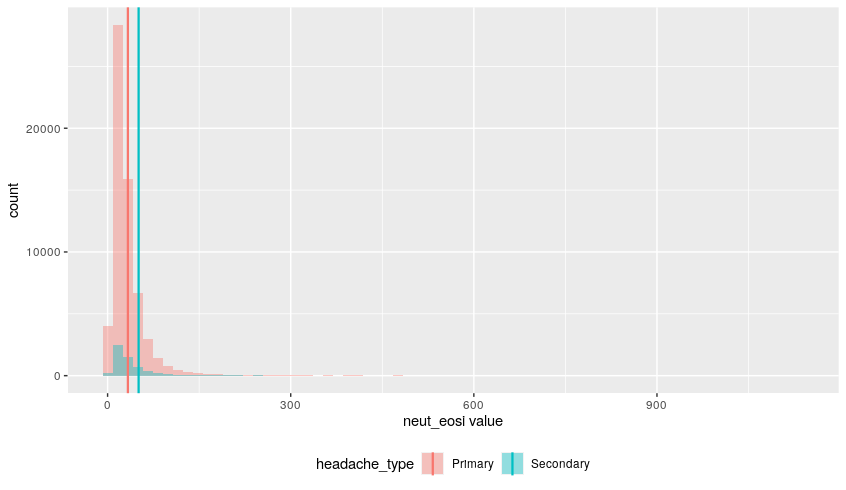


**L**

**M**


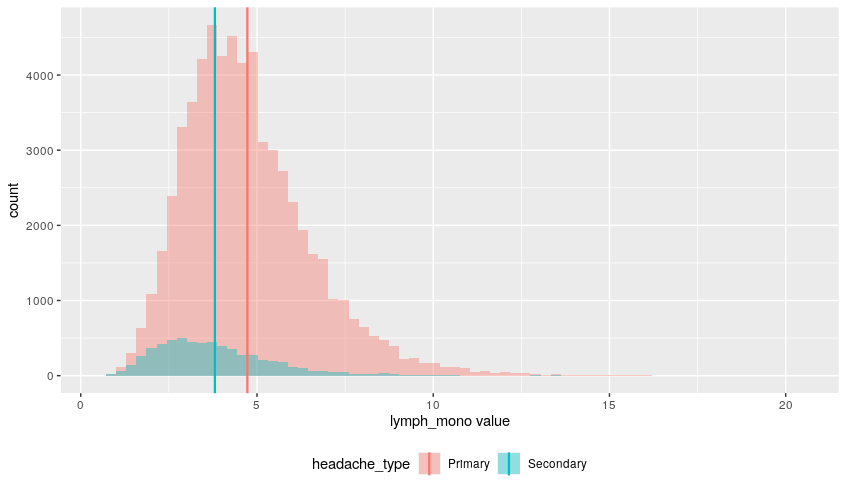


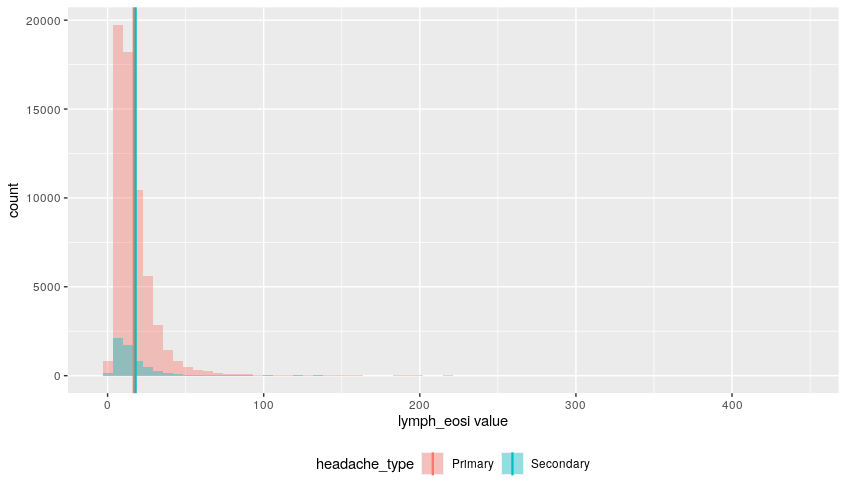


**N**

**O**


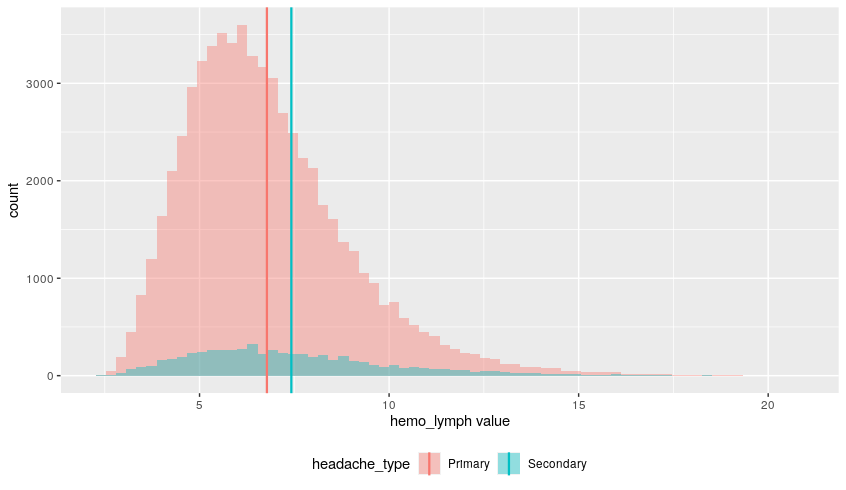


**P**


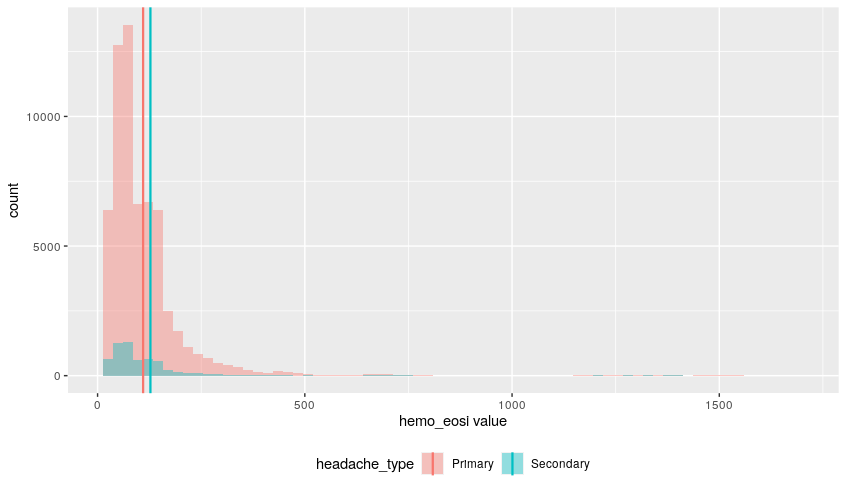


**Q**


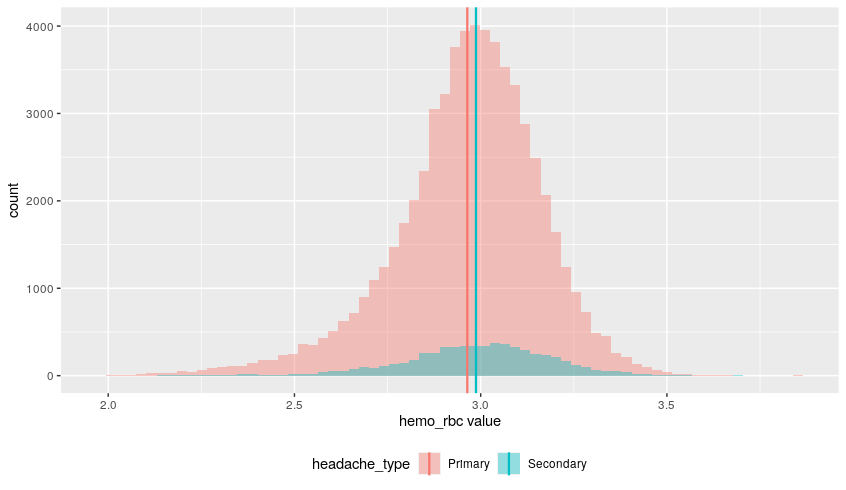


**Q)**


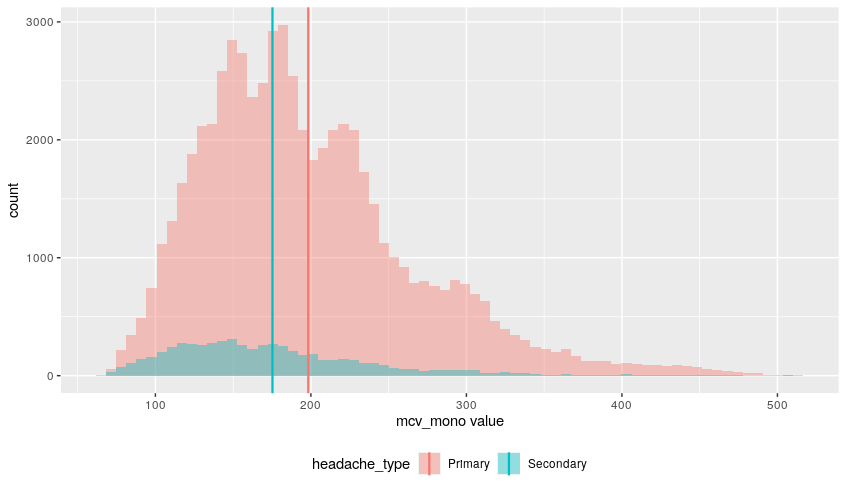


**R**

**S**


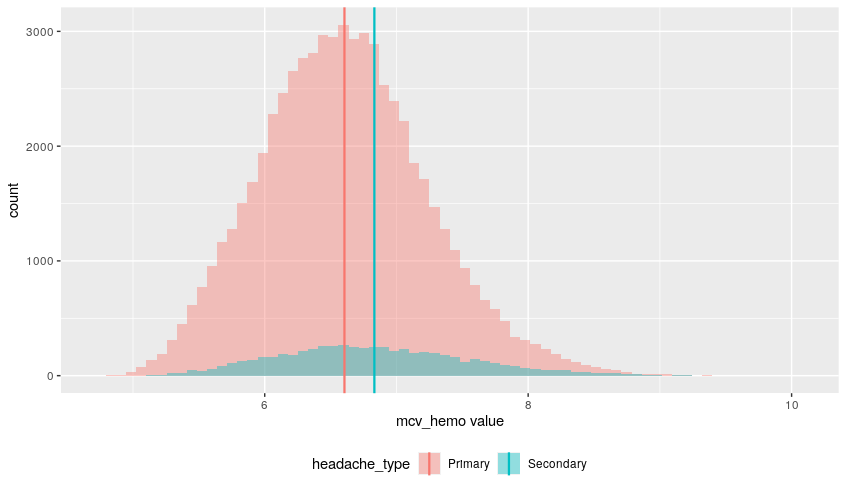

Supplement: S2 Fig — Distribution by headache group of the following ratios: A. platelet/RBC, B. WBC/RBC, C. RBC/neutrophil, D. RBC/monocyte, E. monocyte/eosinophil, F. platelet/MCV, G. platelet/lymphocyte, H. platelet/eosinophil, I. MCV/WBC, J. MCV/neutrophil, K. neutrophil/lymphocyte, L. neutrophil/eosinophil, M. lymphocyte/monocyte, N. lymphocyte/eosinophil, O. hemoglobin/lymphocyte, P. hemoglobin/eosinophil, Q. hemoglobin/RBC, R. MCV/monocyte and S. MCV/hemoglobin. Solid vertical lines indicate the mean of the distribution. MCV, mean corpuscular volume; RBC, red blood cell; WBC, white blood cell. (DOCX) [file pone.0282237.s012.docx]

## **S3 Fig.**


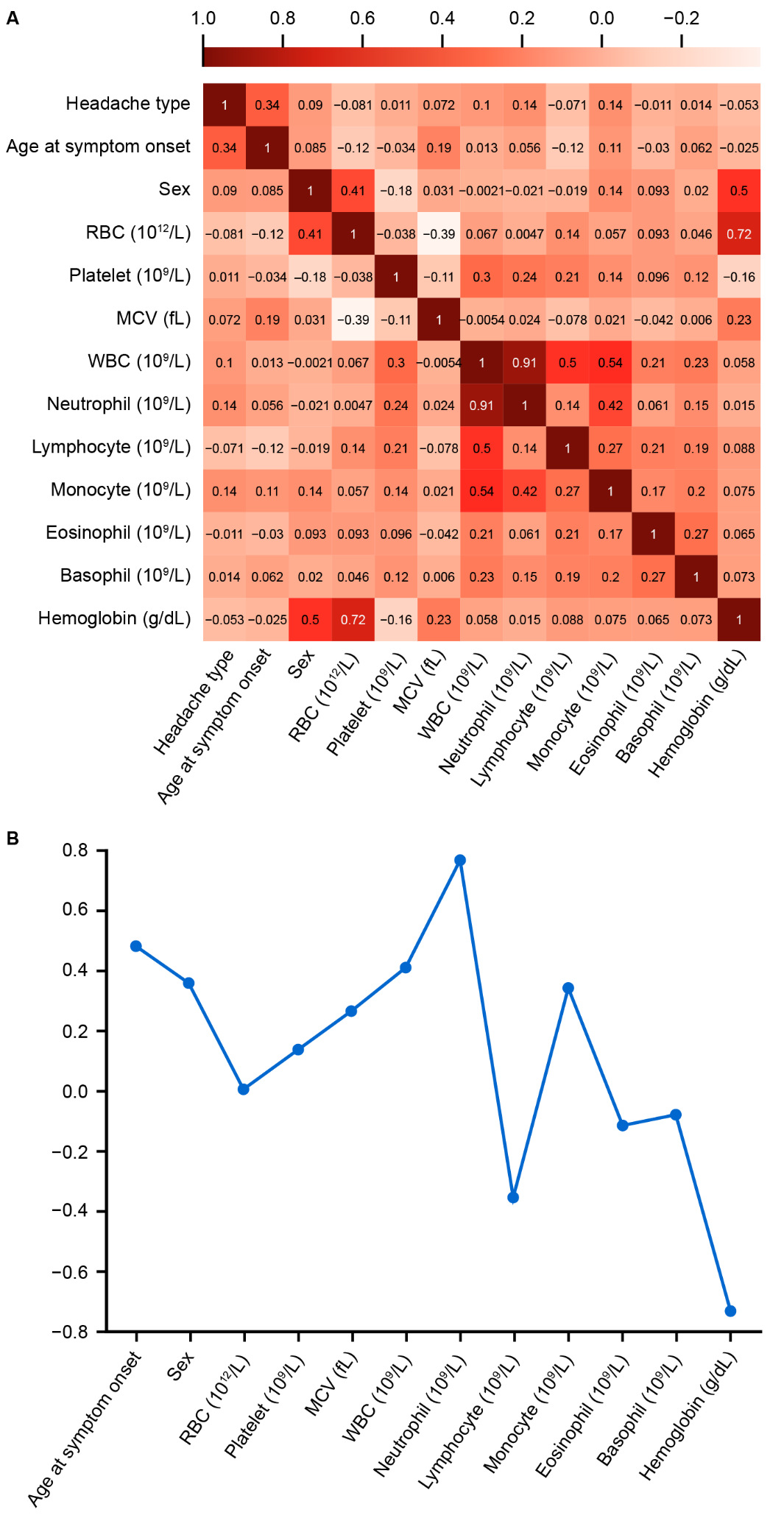

Supplement: S3 Fig — A. Spearman’s correlation matrix and B. feature weight analysis for the features age group, sex and 10 variables from the CBC test, derived from the logistic regression model. CBC, complete blood count; MCV, mean corpuscular volume; RBC, red blood cell; WBC, white blood cell. (DOCX) [file pone.0282237.s013.docx]
